# Indents

**Published:** 1908-03-15

**Authors:** 


### INDENTS.

☞ **Brief Articles of Technical or Scientific Value will receive notice and comment. Contributions invited**

**British Odontological Society.** A meeting of British dentists held a meeting in London, England, and formed a Society for the "Study of Orthodontia."

**For Catarrh.** R. Tincture of ecchinacea, ℥ j.
Compound syrup of stillingia, ℥ v.

Take one teaspoonful every 4 hours for acute catarral inflammations.—Dr. A. Z. Caple in *Ellingwood's Therapeutics.*

**To Clean Impression Trays.** Put the trays and a piece of Sal Soda the size of a walnut in a half gallon of water and boil for fifteen minutes. Take out trays, and wipe dry while warm.—*Dental Cosmos.*

**Articulating Paper.** Instead of using carbon articulating paper, which is extensively employed to locate the high points on masticating surfaces of artificial teeth, I use a piece of typewriter ribbon, which has given better results.—W. H. TAGGART, CHICAGO, in *Den al Review.*

**Primitive Dentistry.** The day is surely coming when we shall be engaged in practicing preventive rather than reparative dentistry, then we shall so understand the Etiology and Pathology of dental caries that we shall be prepared to combat its effects by systemic medication.—Dr. G. V. Black.

**Pulp Anesthesia.** For pulp anesthesia use a solution of cocaine in glycerine mixed to a thick plastic consistency. If a drop or two of adrenalin chloride is added the operation will be bloodless. Glycerine is a strong solvent for cocaine and its strong affinity for water makes it readily absorbed by the pulp tissue. If the pulp is highly congested relieve it first by the use of a refrigerant, as chloroform or ethel chloride.—T. A. Leach in *Review.*

**Local Anesthetic for Pyorrhoea.**

| | |
|---|---|
| Chloroform | 1 ounce |
| Cocain | 20 grains |
| Oil of Cloves | 8 drops |
| Oil of cassia | 8 drops |
| Menthol | 8 grains |

Before removing deposits from roots, saturate a pellet of cotton with this solution, crowd it gently into pockets and allow it to remain a few moments. Be careful to keep cork in bottle, as the chloroform will evaporate rapidly.—*Brief.*

**Investment Material.** Mr. George Brunton, Leeds, England, recommends the following: Clean white sand two parts, plaster of Paris one part (by measure). Stir them together and mix with a saturate solution of potash alum in water. This is said to set quick, and may be placed in the furnace while wet without risk of cracking or flaking. After soldering it crumbles easily between the fingers.—*British Dental Journal,* October 15, 1907, page 1132.

**Early Crown Work.** Dr. E. F. Day, of London, England, showed at the meeting of the American Dental Society of Europe, a porcelain crown that was placed in a ladies' mouth in May, 1851. The root was an incisor and the crown was made of an English tube tooth set on a gold pin set into the root canal. There was no cap over the end of the root. The face of the crown had been ground to imitate the adjacent teeth, on which the enamel had formed imperfectly.

**Administering Gas.** It is not necessary to diet before the administration of gas. The position of the patient is of the greatest importance, and in operating the head should be well erect, so that tooth substance or blood clots do not get into the throat, and thereby cause strangulation. In regard to requesting patients to loosen their clothes, I find it is not necessary unless the patient feels uncomfortable, in which case the collar may be loosened.—A. B. Allen, in *Review*.

**Proteol.** P. Vanel of the Paris Ecole Dentaire, recommends this substance as a root dressing. Proteol is a combination of casein and formic aldehyde, and for dental purposes is to be had in the form of a fine white powder. It is mixed with alcohol and engenol to a suitable paste and packed into the canal. The author has had but two unsuccessful treatments in about fifty cases. The formic aldehyde is the effective ingredient as it is highly antiseptic.—*December Dental Cosmos*.

**Death from Chloroform.** Mrs. Mary Jordan died here this afternoon in a dentist's chair while having eleven teeth extracted. Mrs. Jordan was placed under the influence of chloroform, administered by Dr. C. C. Cobleigh, and R. C. Graham, a prominent dentist, extracted the teeth. After it was over Mrs. Jordan did not revive, and it was revealed that she had died during the operation. Dr. Cobleigh states that Mrs. Jordan did not have a weak heart, as far as his examination revealed.—*Nashville American*.

**Non-Cohesive Foil for Filling.** Do not overlook the value of non-cohesive foil for filling teeth, not in little pellets, but in rolls containing never less than one sheet and sometimes two or three. As a method it is quick and sure. I would prefer to use non-cohesive foil in proximal cavities of bicuspids with cohesive foil in the step, but I would not advise its use always to the exclusion of the inlay. Where an inlay is the best method use it, meaning always the cast inlay, and the very best one you can make.—George W. Haskins, in *Review*.

**Pulp Removal.** If a tooth can be prepared from a mechanical aspect so as to admit of the accurate adjustment of an artificial crown, and if such preparation does not seem to endanger the vitality of the tooth, then to devitalize the pulp would perhaps be unnecessary, and consequently wrong, but unless this may be done in a thorough and conscientious manner—which is seldom possible—then devitalization becomes an absolute necessity, and must be resorted to whether we believe in it or not.

**Lead Points in Root Filling.** Dr. Day, of London, England, in the February *Review*, relates several cases of chronic alveolar abscess, which he had successfully cured by filling the roots with lead points, in some cases allowing them to project through the ends of the roots. He finds this treatment particularly effective in enlarged opical openings, caused by long-standing putrescent conditions. The usual disinfectant treatment is carried out, and then a paint made of metallic lead is introduced tightly into the canal.

**Prevention of Tooth Decay.** Low discusses briefly the report of the committee on scientific research of the New York State Dental Society, in which is recommended the administration of potassium sulphocyanate in one-grain doses, before retiring at night, for the prevention of tooth decay. This commission found that in mouths where rapid and general decay of the teeth exists, potassium sulphocyanate is absent, whereas this salt is present in large quantities in the mouths of individuals whose teeth are immune to decay.—*Buffalo Medical Journal.*

**A Local Anesthetic.** R. Cocain,

Phenol,

Camphor,

Chloral, aa grs j.

Distilled water, ʒ iij.

Rub the Camphor, Phenol and Chloral together in a warm mortar, then dissolve the cocaine in the water and add it to the other ingredients, when thoroughly mixed by aggitation, filter. It may be used locally by rubbing it on the mucous membrane or injected into the gum hypodermatically.

**Dentistry in the French Army.** The French under-Secretary of State for War has recently issued a circular regarding the dental hygiene of the army. Up to the present time this matter has met with very little attention in the army, in spite of the fact that dental lesions have been responsible for a large number of days of absence through sickness; thus, in 1903, they caused 1,845 soldiers to enter hospital, whose days of treatment amounted to 18,639. In the future the School of Hygiene at Val de Grace will give a course of instruction in stomatology. When recruits are enrolled the examining surgeons will make special reports on the state of the mouth and teeth of each soldier, which will be kept up to date by a three-monthly examination. This new system will not only save a number of days of sickness amongst young soldiers in the future but it will instil into them habits of hygiene which they may keep up after they have left the colours and which they may spread around them to the public welfare.—*Dental Record* .

**Death After Nitrous Oxide.** Mr. Walter Schroeder held an inquest at Holborn on Louisa Allen, 30, who died after a dental operation.

The husband, Alexander Allen, a Hanwell milman said his wife had suffered from neuralgia for some time, and had been under medical treatment for the last three months. Acting on advice, she went away for three weeks, but the neuralgia still remained. Next she went to the Welbeck Street dispensary for epilepsy and paralysis, and she was advised to consult Mr. Cox-Moore regarding her teeth. She went to him and he extracted four of her teeth. She left home again seemingly in good spirits, in order to go and have more teeth taken out. Later in the day witness was summoned by telegram, and on arriving at the dentist's he found that his wife had died under the operation.

Mr. Edward Cox-Moore said he had practised as a dental surgeon for twenty-five years. Mrs. Allen went to him as a Hospital Saturday Fund patient, and had four teeth extracted under nitrous-oxide gas anæsthetic. He gave no instructions as to what meals she was to have before coming. When she came on the second occasion he administered a moderate dose of nitrous oxide, using a face apparatus which was regarded as perfectly safe. In fact, it was the best known method The gas took effect, and he took out the teeth that had been selected, and found the woman in a normal state and regaining consciousness. About two minutes later she lurched forward and died. He believed that the patient was not under the influence of the anæsthetic when she died, although he would not positively state that she had recovered consciousness. He had for the last seven or eight years given an anæsthetic to about 1,500 persons annually, without mishap.

Witness's statement was corroborated by John L. Burden, who assisted him.

The evidence of Dr. W. Harper, Bloomsbury Square, was to the effect that death was due to syncope whilst suffering from fatty degeneration of the heart and whilst under the influence of an anæsthetic. This evidence was confirmed by the police surgeon, Dr. Rose, who was present during the post-mortem examination.

A verdict of "Death from misadventure" was returned. —*Dental Record.*

**Proprietary Remedies.** In a recent number of the Journal of the American Medical Association, an article upon the "Bactericidal Value of Some Widely Advertised Antiseptics," by Drs. Verhoeff and Ellis, of Boston, attracted my attention, and I thought it might be of value to the members of the Institute to get a short resume of what they have done.

This article pointed out that if Hydrocele fluid is added in sufficient amount to the various antiseptic silver solutions, the bactericidal power is immediately destroyed. Thinking that this inhibitory action of serum in all probability held good in all other non-irritating antiseptics, these men decided to investigate. After experimenting with certain of the commonly known proprietary antiseptics, choosing those that seemed representative the following results were obtained:

Antiseptics acting alone in the strength specified, failed to kill the staphylococcus aureus, after an exposure of over four hours:

| | |
|---|---|
| Alkalol | 100% |
| Chrystalline | 100% |
| Alkathymol | 100% |
| Glycothymoline | 100% |
| Vaginal Antiseptic (Abbott's) | 2.5% |
| Borol | 50% |
| Cuprol | 5% |
| Zinc sulpho carbolate | 1% |

The strength of the solution te ed was in every case not less than that recommended as effective by the makers.

It was concluded that the above-named solutions were not more effective than a normal salt solution and not nearly so inexpensive.

The following antiseptics acting alone were more or less effective, but when mixed with an equal volume of Hydrocele fluid, failed to destroy the vitality of the staphylococcus pyogenes aureus after an exposure of over two hours:

| | |
|---|---|
| Liquor Antisepticus | 100% |
| Listerine | 100% |
| Lysol | 1% |
| Cresylone | 1% |
| Trikresol | 9–10% |
| Acetozone | 1–1000% |
| Alphazone | 1–1000% |
| Euthymol | 100% |

There were others not met with so often, where the antiseptic action required from five minutes to two hours.

While this is far from all the antiseptics, those named contain the active principles of all on the market, and so are representative. If we were to give our patient a prescription for the Liquor Antisepticus, U. S. Phar., we would doubtless secure a remedy the equal or superior ot any proprietary article on the market, we could know exactly what we were prescribing, and the material is much less expensive than the proprietary article.—H. L. Wheeler in *The Journal.*

**Pharmacopeal Drugs.** I would like to emphasize the importance of accuracy in our prescriptions and the use of Pharmacopeal preparations in place of proprietary drugs. The preparations mentioned can be obtained anywhere, and at considerably less expense than the proprietary articles:

The first is Liq. Antisepticus.

| | |
|---|---|
| Boric Acid | 20.00 |
| Benzoic Acid | 1.00 |
| Thymol | 1.00 |
| Eucalyptol | 0.25 |
| Oil Peppermint | 0.50 |
| Oil Gaultheria | 0.25 |
| Oil Thyme | 0.10 |
| Alcohol | 250.00 |
| Water, qs | 1000.00 |

This makes a preparation slightly acid in reaction, it is pleasant to the taste and is a reasonably active germicide. It can be retailed with profit for about 50 cents for 16 ounces. Listerine, which it resembles, costs about 75 cents for 14 ounces. The patient has been saved expense and you have the added advantage of not using a proprietary article.

There are one or two other preparations to which I would like to call attention. The first of these is Cataplasma Kaolini. This is a preparation similar to Antiphlogistine, is designed for external application, can be obtained anywhere, and has the advantage of being a standard preparation.

If we are going to employ an analgesic, let us do so with a full knowledge of what we are prescribing, and not resort to the use of such proprietary preparations as antikamnia phenalgin, ammonol, etc. A standard preparation of this nature can now be obtained known as acetanlid compound containing acetanlid, 70 parts; bicarbonate of soda, 20 parts; citrate of caffeine, 10 parts. In its use one knows precisely what he is prescribing; the cost is about 35 cents per ounce instead of $1.00, the cost of proprietary preparations of a similar nature.

Acetphenetidin is now the official name of phenacetine and should be employed in prescribing it. The patent on this drug expired about a year ago, at which time it was sold at wholesale for $1.00 per ounce. It can now be obtained for less than half that amount.

In the new pharmacopoeia, the patent tinctures are now 10%. Tincture of aconite is now 10% instead of 35%. If in writing a prescription one wants the 35%, it must be specified.

It is incumbent upon us to employ standard preparations. Let us discourage the use of too many proprietary articles, which are more or less worthless, and impose not only extra expense upon our patients, but preparations which are often of inferior quality.—Dr. A. H. Merritt in *The Journal.*

**Dental Surgery in Antiquity.** In a recent number of the *Rivista Italiana di Odontoiatrica* Professor Galli states that, on carefully examining a skull dug up in the necropolis of an Etruscan city, he found marks of old dental work in the shape of four golden capsules or crowns; two of these covered natural teeth, and had the other two as a bridge between them. Galli is convinced that his discovery shows the work of ancient Etruscan dentists; they also filled decaying teeth very well, and their crowns and bridge work had stood the test of ages. This discovery, though interesting, is not original. Signor Ernesti Mancini contributed to the *Nuova Antologia* of July 16th, 1905, an illustrated article on dentistry in antiquity, to which reference was made in the *British Medical Journal* at the time. Cicero, in his treatise *De Natura Deorum*, ascribes the invention of tooth-drawing to Aesculapius, third of the name. The first mention of dentistry is found in Hippocrates, who in several parts of his writings has a good deal to say about toothache. Long before the dawn of Greek civilization, however, dentistry seemed to have reached a high degree of perfection. From the Phœnicians the art found its way to the Etruscans. At the International Congress held in Rome in 1900 Professor Guerini exhibited several specimens of dental art which proved that something very much akin to bridge work was practised in ancient Italy so efficiently that it lasted thirty centuries. Artificial crowns have also been found in Etruscan tombs. Artificial dentures go back to a remote antiquity. Dr. Deneffe states that in the museum of the University of Ghent there is a set of artificial teeth found in a tomb at Orvieto with jewels and Etruscan vases; he gives their date as from five to six thousand years before Christ. In a collection of antique surgical apparatus made by Dr. Lambros there is an artificial denture found in a tomb at Tanagra, near Thebes, which is believed to belong to the third or fourth century before the Christian era. Teeth stopped with gold have been found in Greek tombs. In the Temple of Apollo at Delphi there was, according to Erasistratus, a nephew of Aristotle and physician to Seleucus Nicator, King of Syria, *circ.* 354 B. C., a leaden instrument which was used in the extraction of teeth; obviously an instrument made of lead could have been used only for loose teeth. In the Laws of the Twelve Tables made by the Roman Decemvirs in 450 B. C. it was expressly forbidden to bury or burn gold with dead bodies, except when used for wiring the teeth. In the construction of false teeth recourse was had by the ancients to bone and horn; sometimes human teeth were employed. Benzoni found in some mummies artificial teeth made of sycamore. In the first century of our era false teeth were very common among the Romans. The name of a fashionable dentist is enshrined in a line of Martial:

Eximit aut dentem Crescentius aegrum.

Elsewhere he ungallantly twits a lady with removing her teeth as she takes off her clothes at night. He says rude things of the teeth of two other ladies:

Thais habet nigros, niveos Licania dentes,

Quae ratio est? Emptos haec habet, illa suos.

Dentistry shared in the decay of the arts during the Middle Ages, and we read that when St. Louis died in 1270, although he was only 55, he had but one tooth in the upper jaw. French surgeons, notably Ambroise Pare, took a leading part in the revival of dentistry. Louis XIV.'s dentist used only instruments of gold in operating on the teeth of his august patient. From the time of Pare onwards the higher dentistry was in the hands of surgeons, extraction being left to the barbers and quacks. Artificial dentures in the sixteenth and seventeenth centuries seem to have been intended rather for show than for use. It is recorded of Mdlle. de Gournay—Montaigne's *fille d'alliance*—that she took out her teeth when she wished to eat, and replaced them when she wished to talk. It is surprising to hear that the humble, necessary toothbrush did not come into general use till towards the end of the eighteenth century.—*British Medical Journal.*

